# Changes in Autonomic Balance, Cardiac Parasympathetic Modulation, and Cardiac Baroreflex Gain in Older Adults Under Different Orthostatic Stress Conditions

**DOI:** 10.3390/healthcare13192404

**Published:** 2025-09-24

**Authors:** Dihogo Gama de Matos, Jefferson Lima de Santana, Felipe J. Aidar, Stephen M. Cornish, Gordon G. Giesbrecht, Albena Nunes-Silva, Roman Romero-Ortuno, Todd A. Duhamel, Rodrigo Villar

**Affiliations:** 1 Cardiorespiratory & Physiology of Exercise Research Laboratory, Faculty of Kinesiology and Recreation Management, University of Manitoba, Winnipeg, MB R3T 2N2, Canadasantanaj@myumanitoba.ca (J.L.d.S.); 2 Graduate Program in Physical Education and Kinesiology and Physiological Sciences, Federal University of Sergipe, Sao Cristovao 49100-000, Brazil; 3 Research Associate, Centre on Aging, University of Manitoba, Winnipeg, MB R3T 2N2, Canada; stephen.cornish@umanitoba.ca; 4 Faculty of Kinesiology and Recreation Management, University of Manitoba, Winnipeg, MB R3T 2N2, Canada; 5 Departments of Anesthesia and Emergency Medicine, University of Manitoba, Winnipeg, MB R3T 2N2, Canada; 6 Laboratory of Inflammation and Exercise Immunology, Department of Physical Education, School of Physical Education, Federal University of Ouro Preto, Ouro Preto 35400-000, MG, Brazil; 7 Discipline of Medical Gerontology, School of Medicine, Trinity College Dublin, D02 PN40 Dublin, Ireland; 8 Institute of Cardiovascular Sciences, St. Boniface General Hospital Albrechtsen Research Centre, Winnipeg, MB R3T 2N2, Canada; 9 Department of Physiology and Pathophysiology, University of Manitoba, Winnipeg, MB R3T 2N2, Canada

**Keywords:** heart rate variability, cardiac parasympathetic modulation, baroreceptor gain, older adults, active standing

## Abstract

**Background**: As we age, our autonomic function declines, resulting in altered autonomic balance during postural transitions. These changes can affect the dynamic interplay between sympathetic and parasympathetic modulation, compromising short-term compensatory responses to active standing. **Objectives**: This study aimed to compare heart rate variability (HRV) at baseline, cardiac parasympathetic modulation (CPM), and cardiac baroreflex gain (CRG) between younger adults (YA) and older adults (OA) following active standing orthostatic stress. A secondary objective was to analyze the incidence of orthostatic intolerance (OI) symptoms. **Methods:** Participants (*n* = 76) completed sit-to-stand and lie-to-stand maneuvers with continuous beat-to-beat blood pressure and heart rate (HR, electrocardiogram). HRV at baseline was analyzed in both time and frequency domains. CPM was measured by the HR 30:15 ratio on standing. CBG was determined as the ratio of HR and SBP changes (ΔHR/ΔSBP) at specific phase time points (30 s, 60 s, 180 s, and 420 s). **Results**: At baseline, OA showed reduced Standard Deviation of RR intervals (SDRR), Root Mean Square of Successive Differences (RMSSD), low-frequency (LF), and high-frequency (HF) power, and elevated LF/HF ratio (all *p* < 0.05), indicating a shift toward sympathetic dominance. During active standing orthostatic stress, OA demonstrated a lower HR30:15 ratio and CBG in later phases (phases 2–4) (all *p* < 0.05). Also, OA reported more symptoms (14%) of OI than YA (0%) (*p* = 0.041). **Conclusions**: These findings indicate that older adults have impaired autonomic function characterized by reduced HRV, CPM and CBG responses. These impairments lead to diminished autonomic regulation under active-standing orthostatic stress and a higher incidence of OI symptoms.

## 1. Introduction

Transitioning from supine or sitting to a standing upright posture is marked by a rapid, transient drop in blood pressure (BP), generating a stimulus for the autonomic nervous system (ANS) to compensate for this BP drop [1,2]. This hemodynamic challenge activates compensatory mechanisms, including an increase in sympathetic activity and a reduction in parasympathetic tone [3]. These responses facilitate vasoconstriction and an increase in heart rate (HR), contributing to the rapid short-term compensatory BP response and maintenance of cerebral perfusion and brain blood flow [4,5].

As we age, the ANS undergoes a gradual decline that impacts its response during postural transitions [6]. This age-related autonomic change is characterized by compromised baroreceptor sensitivity to activate the sympathetic nervous system [7]. Also, the decline in vagal tone further limits the rapid withdrawal of parasympathetic influence necessary for a timely HR increase [5]. Additionally, decreased responsiveness of adrenergic receptors in the heart and vasculature contributes to a less effective sympathetic output [7]. Changes in chemoreceptor function and a decline in muscle pump further contribute to the inability to counteract orthostatic stress [5,7]. A delayed onset of these mechanisms can result in prolonged hypotension during postural transitions, elevating the risk of dizziness, lightheadedness, altered vision from retinal hypoperfusion, and falls [5].

Heart rate variability (HRV) is a key marker of autonomic balance that reflects the interplay between sympathetic and parasympathetic activity [8]. In older adults, HRV is generally reported to be reduced, indicating diminished parasympathetic modulation and impaired autonomic response [8]. Although studies suggest a decline in HRV with aging [9,10], the literature remains inconclusive, as some findings indicate that HRV parameters, such as root mean square successive difference (RMSSD) and standard deviation of R-R intervals (SDRR), may be preserved in healthy older individuals [11,12].

Another factor related to older adults is the deterioration of the cardiac parasympathetic modulation (CPM), in which the ∆HR 30:15 ratio can be used as a marker of this deterioration [13]. Despite its established relevance in assessing autonomic function [13], its role in BP regulation during active standing orthostatic stress has not been thoroughly investigated in older adults. To date, no studies have specifically examined autonomic responses through CPM analysis during active standing using a lower gravitational stress (sit-to-stand) and a higher gravitational stress (lie-to-stand), comparing older adults with younger adults.

As we get older, the function of baroreceptors, primary sensors for BP fluctuations, becomes impaired, which significantly contributes to the overall reduction in autonomic responses [14]. Evidence has shown that, in older adults, BP regulation can be challenging during standing due to the decline in baroreceptor sensitivity (stretch receptors located in the aortic arch and carotid sinus) and cardiac baroreflex gain [14,15,16]. Although existing literature has emphasized the key role of baroreflex in hemodynamic regulation, studies have yet to investigate their response across specific time intervals following active standing. To address this gap, our study analyzed cardiac baroreflex gain (CBG) in four critical phase-specific time points: 30 s (phase 1), 60 s (phase 2), 180 s (phase 3), and 420 s (phase 4), to determine whether the ANS can regulate BP (indicating preserved function) and how it compensates when function is impaired.

This study primarily aimed to **(1)** describe and compare HRV at baseline, **(2)** assess ∆HR 30:15 response immediately on standing, as a surrogate of CPM, and **(3)** evaluate CBG responses at 30 s (phase 1), 60 s (phase 2), 180 s (phase 3), and 420 s (phase 4): when transitioning from (1) sit-to-stand (lower orthostatic stress challenge) and (2) lie-to-stand (higher orthostatic stress challenge). Secondarily, it also aimed to **(4)** compare the number of OI symptoms between younger and older adults. We expected that: compared to younger adults, older adults will show **(i)** a lower HRV (time and frequency domains); **(ii)** lower ∆HR 30:15 values (CPM) on standing; (**iii**) reduced CBG, particularly at 30 s (phase 1) and 60 s (phase 2), but no differences are anticipated at 180 s (phase 3) and 420 s (phase 4). At this point, the CBG responses are sufficient to stabilize hemodynamic parameters. **(iv)** Older adults will report a higher incidence of OI symptoms than younger adults. The observed responses in older adults can be attributed to age-related deterioration in autonomic regulatory mechanisms, an imbalance between sympathetic and parasympathetic influences, reduced CPM and baroreceptor-mediated autonomic control, resulting in a higher incidence of OI-reported symptoms.

## 2. Materials and Methods

### 2.1. Study Design

This was an observational cross-sectional study that adhered to the Strengthening the Reporting of Observational Studies in Epidemiology (STROBE) [17] and the Sex and Gender Equity in Research (SAGER) [18] Reporting guidelines.

### 2.2. Settings

This study took place at the Applied Research Centre within the Faculty of Kinesiology and Recreation Management at the University of Manitoba between August 2022 and August 2023. Ethical approval was granted by the University of Manitoba Health Research Ethics Board (approval number—HE2022-0058). All participants read and signed the informed consent form before participating in the study.

### 2.3. Participants

A convenience sampling strategy was employed to recruit participants. Older adults were drawn from Dr. Todd Duhamel’s database at the University of Manitoba, which comprises ~1000 females who previously participated in the Women’s Advanced Risk-assessment in Manitoba (WARM) Hearts study, as well as from the Centre on Aging database (University of Manitoba), and in-person visits to Men’s Shed and retirement or care homes in Winnipeg. For younger adults, recruitment was carried out by telephone, email, social media platforms (including Facebook, Instagram, and X), advertisements, flyers, posters, and word of mouth.

The sample included both male and female participants divided into two distinct age groups: younger adults (18–30 years) and older adults (60–79 years). Eligibility required that individuals be in these specific age ranges and identify their sex at birth as either female or male. Participants with a history of cardiovascular conditions, such as ischemic heart disease, acute myocardial infarction, stroke, percutaneous coronary intervention, coronary artery bypass surgery, congestive heart failure, psychiatric disorders, and severe cognitive impairment, or who were pregnant, were excluded from the study.

**Figure 1:**
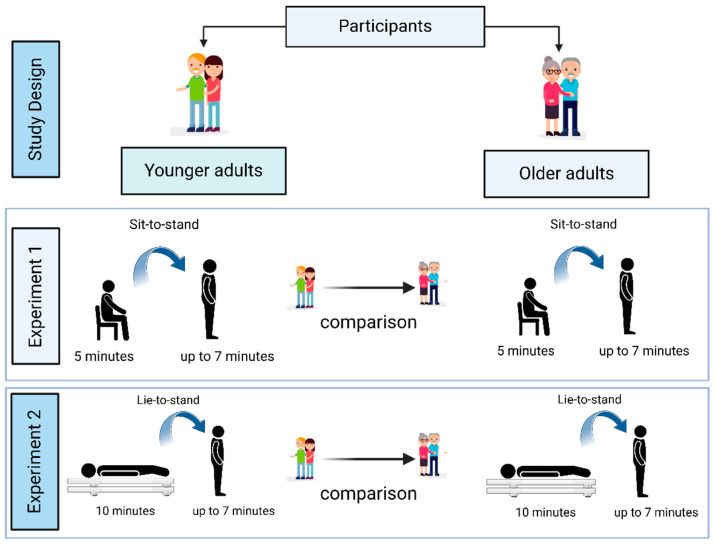
Illustration of the research design and experimental conditions (1 and 2).

Eighty-eight participants underwent orthostatic stress challenges in two experimental conditions: sit-to-stand (Experiment 1) and lie-to-stand (Experiment 2) (Figure 1).

### 2.4. Data Collection

Participants were given preparatory instructions 48 h before their appointment. Upon arrival, they read and signed an informed consent form. Before the assessments, a trained researcher measured resting HR and BP to verify that each participant met the safety criteria outlined by the Canadian Society for Exercise Physiology (CSEP) guidelines [19].

After obtaining informed consent and completing familiarization procedures, participants performed two active standing tests (sit-to-stand and lie-to-stand) with BP measured continuously on a beat-to-beat basis using a Finometer^TM^ Pro Device (Finapres Medical System BV, Amsterdam, The Netherlands). Immediately following the test, participants were asked about any symptoms such as dizziness, light-headedness, and nausea to assess orthostatic intolerance (OI). All assessments were conducted in a quiet room maintained at 22.0 ± 1.0 °C with humidity and barometric pressure at ambient. Testing occurred on weekdays between 8:30 am and 11:30 am to minimize circadian cycle influences. The order of the sit-to-stand and lie-to-stand conditions was randomized and counterbalanced among participants. Medication use was documented through participant self-report but was not included as a control variable in the analysis. No participants reported current use of hormone replacement therapy. In younger females, the menstrual cycle was not controlled.

#### 2.4.1. Demographic and Anthropometric Measurements

Demographic information, including sex assigned at birth, age, and date of birth, was collected to characterize the sample. In addition, anthropometric and body composition assessments were conducted. Body mass (kg) was measured using an InBody^©^ 270 device (InBody Co., Ltd., Cerritos, CA, USA), and height (m) was measured using a mobile stadiometer (SECA, Frankfurt, Germany).

#### 2.4.2. Assessment of Heart Rate Variability, Cardiac Parasympathetic Modulation, and Cardiac Baroreflex Gain

HRV, CPM, and CBG were obtained by extracting information from the electrocardiogram (ECG) and Finometer (Finapres Medical System, Arnhem, The Netherlands) [20] and recorded using LabChart 8.0 (ADInstruments, Colorado Springs, CO, USA) at a sampling frequency of 1 kHz. A standard three-lead ECG configuration was used, with lead II prioritized for R-peak detection. The ECG signals were subjected to a 5–30 Hz bandpass filter for R peak detection, except during the 30 s before and 90 s after standing, when the raw signal was preserved and Finometer calibration was manually disabled. Cardiovascular parameters during the sit-to-stand and lie-to-stand tests were analyzed with a custom macro for R-R interval detection. The R-R intervals obtained from the ECG served as the basis for HR calculation. Ectopic beats were automatically identified and excluded by the LabChart HRV module and visually inspected, and when necessary, corrected using interpolation to maintain continuity of the R-R interval series. Segments were excluded entirely if >5% of beats required correction, consistent with established HRV preprocessing recommendations [21,22].

Arterial pressure was recorded non-invasively via photoplethysmography at the middle finger of the left hand (Finometer—Finapres Medical System, Arnhem, The Netherlands) [20]. BP calibration was initially performed using the manufacturer’s standard protocol and subsequently refined through manual calibration with an automated BP monitor (ARSIMAI, BSX516, Munster, Germany). Approximately five minutes before each procedure, finger and brachial pressures were adjusted using the return-to-flow calibration. Finger circumference was measured to select the appropriate cuff size. The height correction system was used to adjust the reconstructed BP from the finger arterial pressure wave to the brachial arterial pressure wave (heart) throughout the assessments.

Systolic blood pressure (SBP) and HR data were then stored and transferred to a custom Matlab application (Matlab R2024; The MathWorks Inc., Natick, MA, USA). Once the data were imported from LabChart into the Matlab application, noise and motion artifacts were filtered using a two-step process. Initially, a median filter was applied, followed by a low-pass filter with a cutoff frequency of 0.05 rad/sample, as determined by scalogram analysis. Lastly, a 5-s moving average filter was employed to smooth the signal and isolate the parameters of interest [23,24]. Following data import, interpolation was applied to correct for delays induced by resampling at 1 Hz. The onset of active standing was defined as time zero, with baseline values assigned as negative and positive values representing short-term compensatory responses. When excessive noise or motion artifacts persisted despite filtering and precluded reliable detection of R–R intervals or beat-to-beat blood pressure, the participant’s dataset for that condition was excluded from analysis.

#### 2.4.3. Heart Rate Variability

HRV was obtained by continuous beat-to-beat measurements during supine position using a three-lead electrocardiogram (ECG) module collected at a 1 kHz sampling rate (Finometer, Finapres Medical System, Arnhem, The Netherlands), according to the guidelines from the *European Society of Cardiology and the North American Society of Pacing Electrophysiology* [21]. HRV data were analyzed in time and frequency domains using at least 5 min or a window of ≥256 beats. The HRV data were preprocessed through the HRV module (LabChart 8, ADInstruments, Colorado Springs, CO, USA). The module detects and extracts RR intervals and automatically differentiates between normal and ectopic beats. In the time domain, mean RR intervals, the standard deviation of RR intervals (SDRR), and the root mean square of successive RR intervals (RMSSD) were assessed to evaluate the sympathetic and parasympathetic activity. Spectral analysis of frequency domain variables was estimated using the Fast Fourier transform model. Low-frequency (LF) (0.04–0.15 Hz) and high-frequency (HF) (0.15–0.45 Hz) spectral components were collected in absolute power values (ms^2^). The low-frequency/high-frequency ratio (LF/HF) was calculated from the absolute values of LF and HF.

#### 2.4.4. Cardiac Parasympathetic Modulation

Cardiac parasympathetic modulation (CPM) was measured using the HR 30:15 ratio of the minimum (longest RR-interval) around the 30th heartbeat and maximum HR (shortest RR-interval) around the 15th heartbeat after standing [13]. This ratio was selected because it reflects parasympathetic (vagal) reflex engagement, where tachycardia is maximal around the 15th beat, and relative bradycardia is maximal around the 30th beat [13]. The HR used for the CPM calculation was obtained from the R-R intervals (ECG-module, Finometer, Finapres Medical System, Arnhem, The Netherlands). This measurement is generally used in clinical settings to assess the autonomic cardiovascular reflex function and detect early signs of autonomic dysfunctions [25,26]. It provides insight into the heart’s ability to respond to stress (e.g., postural transition), which could support the diagnosis of conditions that can affect cardiovascular stability, mainly in people with underlying health issues [25,26].

#### 2.4.5. Cardiac Baroreflex Gain

Cardiac baroreflex gain (CBG) was adapted from the proposed method by the Autonomic Disorders Consortium [27] by calculating the change in HR divided by the drop in SBP (ΔHR/ΔSBP ratio), divided into four critical phase-specific time points: 30 s (phase 1), 60 s (phase 2), 180 s (phase 3), and 420 s (phase 4) after active standing, expressed as bpm.mmHg^−1^ [27]. The four time points for CBG assessment (30 s, 60 s, 180 s, and 420 s) were selected based on both clinical definitions of orthostatic hypotension (OH) and physiological expected responses after standing, consistent with the consensus framework [28]. Specifically, 30 s was selected to capture the expected recovery from the initial OH window, 60 s was included as an intermediate checkpoint to assess early stabilization after standing, 180 s corresponds to the consensus definition of classical OH, and 420 s was chosen to capture delayed OH (>180 s) while aligning with the end of our standardized recording protocol. This phase-specific assessment of adaptive CBG responses identifies differences in autonomic regulation during the short-term compensatory responses. The HR used for this calculation was obtained from the R-R intervals (ECG module, Finometer, Finapres Medical System, Arnhem, The Netherlands). SBP was obtained from the arterial pressure non-invasively via photoplethysmography at the middle finger of the left hand (Finometer, Finapres Medical System, Arnhem, The Netherlands) [20]. Calibration procedures (return-to-flow and finger and brachial arterial pressures adjustments), appropriate cuff size, and height correction system adjustments were the same as previously described. CBG assessment is typically used in clinical settings for evaluating BP regulation during active standing, identifying issues with autonomic control that could lead to orthostatic intolerance (e.g., dizziness) [29].

### 2.5. Sample Size

The sample size was determined a priori using the G*Power software (3.1.9.6) (Heinrich Heine University, Düsseldorf, Germany) [30], based on data obtained from an internal pilot with 16 participants and equal distribution between groups. A *t*-test (two independent means) was used to compare the differences in CPM (HR 30:15), with an α level of 0.05. For the sit-to-stand experiment, the analysis indicated a statistical power of 0.81 and an effect size of 1.17, based on mean ± SD values of 0.87 ± 0.06 for younger adults and 0.93 ± 0.04 for older adults, which resulted in an estimated total sample of 20 participants (10 per group). In the lie-to-stand experiment, the estimated power was 0.80 with an effect size of 1.74, using mean ± SD values of 0.82 ± 0.07 for younger adults and 0.91 ± 0.02 for older adults, resulting in a total of 10 participants (5 per group).

### 2.6. Statistical Analysis

Data normality was evaluated using the Shapiro-Wilk test. Variables with a parametric distribution were expressed as mean ± standard deviation (SD) along with the corresponding 95% confidence interval (95% CI), while non-parametric variables were summarized as the median with interquartile range (IQR) and their minimum and maximum values. For sample characterization, continuous data was presented using these descriptive statistics, and categorical data were reported as absolute numbers and relative percentages. Additionally, the Chi-Square test was utilized to assess differences in categorical variables (e.g., number of cases, medication use).

An unpaired Student *t*-test was used to compare differences between younger adults and older adults for both sit-to-stand and lie-to-stand conditions, while the Mann–Whitney U test was applied to non-parametric data. To control for multiple comparisons, *p*-values were adjusted using the Benjamini–Hochberg procedure with a false discovery rate of 0.05, thereby limiting the expected proportion of false positives among significant findings [31]. Significant results were reported based on the corrected *p*-values, highlighting the tests that met the adjusted threshold of *p* < 0.05.

Effect sizes were calculated using Cohen’s *d* for parametric data [32] and by the R coefficient (non-parametric data) [33], with classifications set as low (≤0.05), medium (0.06–0.25), high (0.26–0.50), and very high (>0.50) [32]. All analyses were done using R^®^ (Version 4.1.1, Vienna, Austria).

## 3. Results

**Figure 2:**
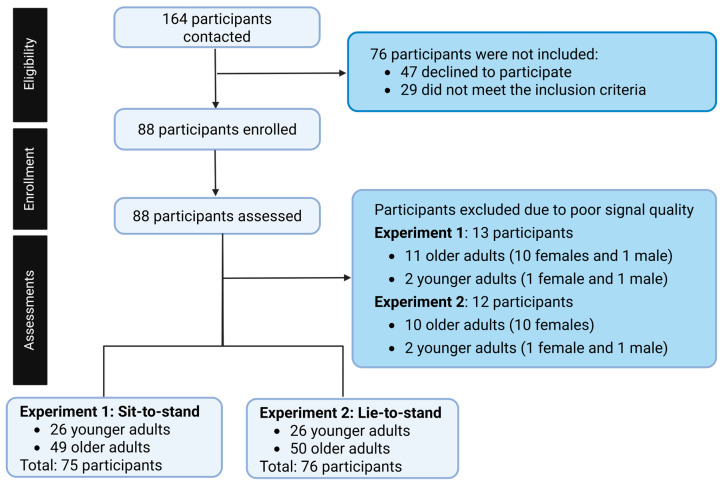
Recruitment flow chart of the study.

Eligibility was assessed using a screening questionnaire developed by our research team to evaluate each participant’s health status. Due to poor signal quality, 13 participants were excluded from Experiment 1 (11 older adults: 10 females and 1 male and 2 younger adults: 1 female and 1 male) and 12 from Experiment 2 (10 older adults: 10 females and 2 younger adults: 1 female and 1 male). As a result, Experiment 1 included 26 younger adults and 49 older adults, while Experiment 2 consisted of 26 younger adults and 50 older adults. Participants excluded from one experiment due to poor signal quality were still included if they contributed valid data in another experiment. Then, their data was retained, and they were included (e.g., demographic characteristics). However, if CPM and/or CBG data quality was poor, participants were excluded from the analysis. Comparisons confirmed that their age, sex distribution, BMI, and baseline hemodynamic measures were not different from the final analyzed sample, suggesting that exclusion was unlikely to introduce systematic bias and affect the data analysis. HRV at baseline, CPM (HR 30:15) immediately on standing, and CBG at 30 s, 60 s, 180 s, and 420 s were compared between younger and older adults in experiments 1 and 2 (Figure 2).

Table 1 summarizes the characteristics of younger adults (18–30 years) and older adults (60–79 years) for sex distribution, anthropometric measurements, the incidence of OI, comorbidity distribution, and medication use. Younger adults had an equal distribution of females and males in both experiments. For experiment 1, 72% of the older adults were female, and 28% were male, whereas in experiment 2, 70% were female and 30% were male. Older adults had a higher body mass index (BMI) and shorter stature compared to younger adults, although no significant differences in body mass were observed. Additionally, older adults were more likely to experience OI than younger adults. The number of cases of hypertension and diabetes, as well as the use of cardiovascular medications, were only present in the older group. Specifically, 14 of the older adults (27%) reported taking calcium channel blockers, 7 (13%) reported use of β-blockers, 5 (10%) use of diuretics, 4 (8%) use of ACE inhibitors, and 4 (8%) use of angiotensin receptor blockers. Psychotropic medications were reported in both groups, with no statistically significant differences between them: antidepressants were used by two of the younger adults (8%) and six of the older adults (12%), and antipsychotics were reported in only one of the older adults (2%). Since the prevalence of specific medications was relatively low (small sample size), we combined medications into broader categories (cardiovascular and psychotropic medications) for statistical analysis (Table 1).

### 3.1. Heart Rate Variability at Baseline in Supine Position

Table 2 presents HRV responses in the time and frequency domains comparing younger adults and older adults at baseline in the supine position. Older adults demonstrated lower SDRR and RMSSD (time domain), lower LF and HF, and higher LF/HF ratio (frequency domain) compared with younger adults. There were no statistically significant differences in RR intervals between groups.

### 3.2. Cardiac Parasympathetic Modulation Responses During Active Standing Orthostatic Stress

**Figure 3:**
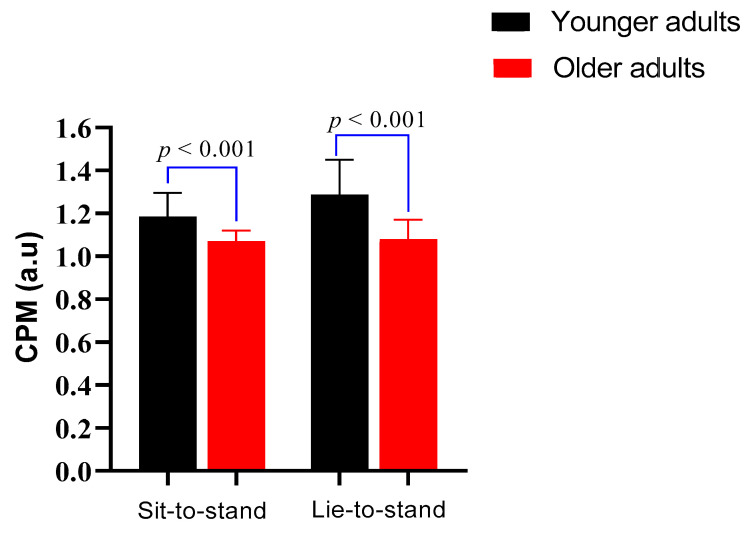
Cardiac parasympathetic modulation (CPM) comparison between younger adults and older adults during sit-to-stand and lie-to-stand transitions. Bars represent median with IQR in sit-to-stand and mean ± SD for lie-to-stand.

Figure 3 shows the CPM comparison between younger adults and older adults during sit-to-stand and lie-to-stand transitions. During the sit-to-stand test (lower orthostatic stress challenge), CPM was reported as median values with interquartile range. Older adults had a significantly lower CPM (1.07, IQR: 0.08) compared to younger adults (1.18, IQR: 0.16) (*p* < 0.001). During the lie-to-stand test (higher orthostatic stress challenge), CPM values were expressed as mean ± standard deviation. Older adults showed lower CPM values (1.08 ± 0.09) than younger adults (1.28 ± 0.16) (*p* < 0.001).

### 3.3. Cardiac Baroreflex Gain Responses During Active Standing Orthostatic Stress Across Specific Phase Time Points

During the sit-to-stand transition, no differences were observed in phase 1 (0–30 s) between the groups. However, older adults showed lower CBG values from phases 24 (30–420 s). In the lie-to-stand, older adults showed lower CBG values in phases 3 (60–180 s) and 4 (300–420 s), but no differences in phases 1 (0–30 s) and 2 (30–60 s) (Table 3).

## 4. Discussion

To the best of our knowledge, this is the first study to analyze together HRV (time and frequency domains), CPM (ΔHR 30:15) and CBG across distinct phase-specific time points comparing older adults and younger adults during two distinct active-standing orthostatic stresses: sit-to-stand and lie-to-stand. Our findings indicate that older adults had reduced autonomic activity as measured through the HRV, lower CPM and CBG from phases 2–4, and higher symptoms of OI.

### 4.1. Heart Rate Variability Responses

In the time domain analysis, there were no statistically significant differences between older adults and younger adults in RR intervals during both transitions. Kawaguichi et al. [34] reported no statistically significant differences in RR intervals at baseline between older adults and younger adults, supporting the results of the current study. Additionally, in this study, older adults exhibited lower SDRR and RMSSD compared to younger adults in both conditions, which corroborated the results of Grassler et al. [35] and Kawaguchi et al. [34].

The reduced SDRR and RMSSD observed in older adults in our study indicate a decline in HRV, which is commonly associated with diminished parasympathetic modulation [8]. Our data shows significantly lower SDRR and RMSSD values in older adults compared to younger adults, supporting the understanding of reduced vagal tone in older adults [36]. This diminished vagal tone is particularly relevant during postural transitions, where parasympathetic withdrawal and sympathetic activation must occur in a coordinated manner to maintain autonomic regulation [8]. These results are consistent with large-scale normative studies. For example, Voss et al. [37] demonstrated pronounced age-related declines in short-term HRV indices. Likewise, Tegegne et al. [38], provided age- and sex-specific reference values for RMSSD, showing a progressive decline across the lifespan in both sexes. Our findings align with these normative benchmarks, confirming that the reduced HRV observed in older adults in our cohort falls with age.

In the frequency domain, our results showed that older adults demonstrated reduced LF and HF power compared to younger adults in both postural transitions, along with an elevated LF/HF ratio. These findings align with those of Grassler et al. [35], who reported significantly reduced LF and HF power in older adults compared to younger adults. Similarly, Kawaguchi et al. [34] also observed that older adults had lower LF and HF compared to younger individuals, along with a higher LF/HF ratio. The reduction in LF and HF power in older adults suggests an overall decline in autonomic activity [8]. A lower LF indicates reduced sympathetic modulation and diminished baroreflex sensitivity, while a lower HF reflects impaired parasympathetic activity [39]. The higher LF/HF ratio observed in older adults points to a shift towards sympathetic dominance and reduced vagal tone. This shift is associated with reduced CPM and CBG and a greater likelihood of OI, as observed in our results.

Unlike previous studies that relied on passive tilt or broad HRV metrics [40,41], our active sitting or lying to standing engages the central command, the vestibulo-sympathetic reflex, and the skeletal muscle pump, providing a closer approximation to everyday orthostatic demands. Additionally, our active stand design allowed the examination of HRV at baseline in relation to subsequent compensatory phases after standing. This approach provides mechanistic insight into how reduced parasympathetic modulation (RMSSD, HF power) in older adults compromises the rapid vagal withdrawal needed for effective autonomic adjustments. Rather than a general reduction in HRV, our findings show that diminished vagal tone at baseline is a precursor to impaired vagal withdrawal that occurs in the initial seconds after standing, which co-occurs with delayed baroreflex-mediated compensation, delaying BP recovery. Through the HRV analysis alongside cardiac parasympathetic modulation (CPM) and phase-specific cardiac baroreflex gain (CBG) responses, we show that lower baseline parasympathetic tone is linked to impaired vagal withdrawal and reduced cardiac augmentation, which will result in a prolonged hypotensive window during these postural transitions. This extends prior passive tilt and generalized HRV work by investigating parasympathetic withdrawal and cardiac augmentation. It is an important note that passive tilting may underestimate deficits that depend on active muscle pump and central command components.

We also focused on HRV analysis under stationary conditions to ensure methodological validity and reproducibility, following Task Force standards and subsequent methodological recommendations [8,21,39]. The immediate post-stand is dominated by baroreflex-mediated vagal withdrawal, sympathetic activation, and marked hemodynamic variability. These rapid fluctuations violate the assumption of signal stationarity required for valid HRV interpretation. Therefore, ultra-short HRV metrics during this window often demonstrate low reproducibility, high inter-individual variability, and limited comparability across studies. Nonetheless, we acknowledge that entropy-based HRV analysis using ECG data [42] and other nonlinear approaches are being actively developed to address the challenge of analyzing HRV during dynamic, non-stationary phases, as the first minute of orthostasis. These methods remain exploratory but may provide valuable complementary markers of transient autonomic reactivity in future studies.

### 4.2. Cardiac Parasympathetic Modulation

This is the first study to compare the ∆HR 30:15 ratio, a validated marker of autonomic cardiovascular reflex function, between older and younger adults. In both conditions, older adults demonstrated a significantly lower HR30:15 ratio compared to younger adults, reflecting an attenuated parasympathetic (vagal) reactivation after standing and a concomitant reduction in timely sympathetic compensatory response around the 30th beat. These age-related autonomic impairments compromise the rapid cardiovascular adjustments necessary to maintain adequate cerebral perfusion, particularly during the early phases of orthostatic stress [4,43]. Together, the reduced ability to promptly coordinate parasympathetic withdrawal, vagal reactivation, and sympathetic activation attenuates HR and vascular resistance responses [5], thereby predisposing older individuals to larger transient declines in BP and an elevated risk of OH and OI [4].

### 4.3. Cardiac Baroreflex Gain Responses Across Specific Phase Time Points During Active Standing Orthostatic Stress

It was observed in this study that older adults exhibited significantly lower CBG compared to younger adults from phases 2 to 4 (30 s to 420 s) during sit-to-stand and from phases 3 and 4 (180 s to 420 s) during lie-to-stand. Evidence consistently demonstrates that older adults have lower CBG compared to younger adults at the fifth minute after a lie-to-stand transition [44], using sequence method analysis [45], and the Valsalva maneuver [46]. In the present study, the observed decline in CBG among older adults during the later phases of both sit-to-stand and lie-to-stand transitions is primarily driven by age-related impairments in baroreceptor function, arterial compliance, and autonomic regulation [14]. Age is associated with increased arterial stiffness, particularly in the carotid sinus and aortic arch, which reduces the mechanosensitivity of baroreceptors and impairs their ability to detect and respond to acute fluctuations in BP [4]. This diminished mechanotransduction results in a blunted baroreflex response, as evidenced by Monahan et al. [14], who demonstrated a strong positive relationship between carotid artery compliance and cardiovagal BRS. Additionally, age-related alterations in neural integration within the nucleus tractus solitarius and reduced vagal efferent responsiveness contribute to delayed and attenuated HR adjustments during postural stress [7]. The findings by Lai et al. [45] and Kim et al. [44] further support this physiological mechanism, highlighting significantly lower BRS in older adults compared to younger individuals.

### 4.4. Symptoms of Orthostatic Intolerance

The comparison of OI symptoms between older adults and younger adults is limited in the literature, making direct age-group analyses challenging. As expected, older adults showed a higher number of OI symptom cases (14%) compared to younger adults (0%). The OI symptoms were statistically different between older adults and younger adults (*p* = 0.041). Since OI was assessed as a secondary endpoint and the number of symptomatic cases was relatively small (*n* = 7), the statistical power for detecting group differences was limited. A posteriori power analysis showed that with our sample size (*n* = 26 younger adults and 50 older adults), we had 51% power to detect a group difference in OI symptoms, corresponding to an effect size of 0.23, at α = 0.05. Therefore, our results need to be interpreted with caution and considered exploratory. Nonetheless, the observed number of cases aligns with previous age-related studies reporting increases in OI [47,48], suggesting that our findings are directionally consistent with the broader literature, even if not definitively powered to establish incidence rates.

Given the scarcity of direct comparisons, the discussion will focus on the physiological mechanisms underlying this difference. The higher OI symptoms indicate an age-related decline in cardiovascular-autonomic regulation. This disparity suggests that older adults have impairments in baroreflex sensitivity [45], reduced parasympathetic modulation [7], and diminished vascular response [43], all of which contribute to compromised hemodynamic stability during postural transitions. The increased susceptibility to OI in older adults may reflect attenuated compensatory mechanisms, including delayed vasoconstriction and inadequate cardiac output adjustments [4,43], leading to transient cerebral hypoperfusion and symptomatic manifestations.

### 4.5. Implications for Older Adults’ Health and Clinical Practice

The findings of this study provide insight into age-related autonomic dysfunction and its relevance to older adults’ health. While the primary focus was on physiological mechanisms, the observed impairments, like reduced HRV, diminished CPM and CBG, and higher incidence of OI, have direct implications for clinical care and healthy aging. These alterations compromise the ability to regulate blood pressure during everyday postural transitions, such as rising from a bed or chair, increasing the risk of dizziness, falls, and death in older populations [1,49].

Our results showed the importance of incorporating beat-to-beat cardiovascular and autonomic assessments into routine geriatric evaluations. For example, using continuous non-invasive blood pressure monitoring during chair rise or tilt-table testing can help detect subtle impairments in compensatory responses that may not be captured with conventional intermittent measurements [2,50]. This approach is particularly relevant for older adults with multimorbidity, polypharmacy, or unexplained falls [2], supporting fall risk, OH, and OI management.

In clinical and rehabilitation settings, our data support the development of individualized interventions and strategies to reduce orthostatic stress (OH and OI). For example, teaching older adults to perform counter maneuvers such as ankle pumps or leg crossing before standing can activate the muscle pump and help enhance venous return and cardiac preload [51,52] during the early phase of orthostasis, which is particularly relevant given the higher incidence of OI symptoms observed in our older participants. Consistent with our finding of delayed parasympathetic withdrawal and reduced baroreflex engagement during the early compensatory phases (CPM results), advising individuals to pause briefly in a seated or semi-recumbent position before rising fully may allow time for autonomic response adjustments to begin before the full orthostatic challenge. Our observation of reduced autonomic regulation in older adults supports recommendations of adequate hydration (e.g., encouraging water intake of ~1.5–2 L/day) and the use of compression stockings in selected individuals as additional strategies to stabilize blood pressure [49,53] due to improvements in central blood volume and buffering capacity against postural hypotension.

Structured exercise programs targeting autonomic improvements, such as moderate-intensity walking, chair-based resistance exercises, or supervised balance training, can enhance baroreflex sensitivity and autonomic function [54]. Follow-up assessments (e.g., re-testing CBG, CPM, HRV, after weeks of exercise) can help clinicians monitor progress and tailor interventions over time [55,56]. Similarly, medication reviews should focus on deprescribing or adjusting agents that blunt autonomic responses, such as certain antihypertensives (e.g., alpha-blockers or vasodilators), where appropriate [57].

Finally, older adults’ education is essential. Providing them with practical information on recognizing early symptoms of orthostatic intolerance (e.g., lightheadedness when rising, blurry vision, fatigue), along with guidance on when to seek medical attention, can empower proactive self-management [2,58]. Simple tools like symptom diaries or blood pressure logs may be used to monitor trends and inform healthcare decisions [2,49].

Translating physiological mechanistic findings into concrete clinical strategies, this research helps bridge the gap between physiological aging and applied geriatric care, supporting functional independence, safety, and quality of life in the growing population of older adults.

### 4.6. Limitations

Some limitations should be considered regarding this study. Although HRV was analyzed using both time- and frequency-domain, these analyses were conducted only at baseline, which may not fully observe the autonomic fluctuations occurring after active standing. The use of short-term or ultra-short HRV analyses may provide additional insights into the dynamic interplay between sympathetic and parasympathetic modulation in response to active standing orthostatic stress. Future studies may incorporate these approaches to offer a more detailed dynamic characterization of autonomic regulation after standing.

CBG was assessed at specific time points (30 s, 60 s, 180 s, and 420 s), which may not fully show the continuous dynamic fluctuations in baroreflex function throughout the active standing orthostatic stress. While this approach provides information on the short-term compensatory responses at critical times during active standing orthostatic stress, it may not account for transient variations or phase-dependent adaptations. Since baroreflex function is inherently a continuously adaptive process, adjusted on a beat-to-beat basis to integrate afferent input and modulate efferent autonomic outflow, our phase-specific approach, while useful for identifying distinct compensatory periods after standing, should be interpreted with caution. This phase-based analysis may underestimate rapid or subtle fluctuations of this dynamic process or miss transient adaptations that occur between the pre-defined windows. Alternative methodologies, such as the sequence method [29], which detects spontaneous changes in SBP and RR intervals to estimate baroreflex sensitivity in real-time, or transfer function analysis [29], which evaluates baroreflex function across different frequency bands, may offer a more comprehensive representation of baroreflex regulation. Future studies applying these continuous or sequence-based methods could complement our findings by providing a more refined characterization of baroreflex engagement during orthostasis. Future work should integrate these methods alongside HRV, CPM, and CBG measures to support building a mechanistically complete picture.

The use of convenience sampling may limit the generalizability of the findings to broaden older adult populations. Participants were primarily recruited through existing databases, local organizations, and community centers, which may have introduced sampling bias by favoring individuals who are more socially engaged or physically mobile. As a result, the sample may not fully represent socially isolated older adults, potentially underestimating the variability in autonomic responses to orthostatic stress within the general aging population. Future studies employing probabilistic sampling methods could help improve external validity and better capture the heterogeneity of responses across diverse subgroups of older adults.

Medication used among older adults was recorded but not controlled, which could have influenced cardiovascular responses during active orthostatic stress. Many older adults take antihypertensives, beta-blockers, or diuretics as previously described, which may affect HR, BP, and vascular resistance [3]. While these medications may have impacted compensatory responses under investigation, they also reflect real-world conditions, making the findings more applicable to the community-dwelling older population. 

In younger female participants, the menstrual cycle phase was not controlled. However, its impact on cardiovascular responses remains controversial in the literature. Studies have suggested that estrogen levels influence vascular tone [4,59] and hemodynamic responses during postural transitions [60,61]. Conversely, other studies report no significant effects on hemodynamic responses [62,63]. Therefore, whether and how the menstrual cycle affected our results, and the extent of this influence, remains uncertain.

Another limitation that should be mentioned is the unequal sex distribution in the older adult group, which had a higher proportion of females. As sex-related differences in autonomic balance are well documented [64,65,66,67], this imbalance may have influenced our findings independently of age. Future studies should aim for more balanced recruitment or stratified analyses to better distinguish the effects of age and sex on autonomic regulation. Sex differences and frailty are potential confounders that should be acknowledged as limitations to the study. Future research should explore how these factors impact cardiovascular responses during active standing orthostatic stress. 

## 5. Conclusions

In conclusion, older adults had greater autonomic dysfunction during active standing orthostatic stress, as evidenced by reduced HRV, impaired cardiac parasympathetic modulation (reflected by a lower HR 30:15 ratio), diminished CBG, and a higher incidence of OI symptoms. These findings indicate that older adults presented autonomic dysfunction, affecting the compensatory mechanisms, leading to a reduced ability to regulate BP and maintain cerebral perfusion during postural transitions. This impairment likely contributes to the higher incidence of OI symptoms in older adults (14%) compared to younger adults (0%), as the diminished autonomic response may result in delayed cardiovascular adjustments, increasing susceptibility to dizziness or lightheadedness on standing. Overall, our findings highlight short-term compensatory autonomic responses impairment in older adults and its potential contribution to orthostatic intolerance, with important implications for fall risk prevention, geriatric care, and rehabilitation.

## Figures and Tables

**Table 1:** Sample characteristics of younger adults and older adults.

Variable	Younger Adults			Older Adults			*p*-Value
**Sex**	*n*	%	-	*n*	%	-	-
*Experiment 1*	*-*	*-*	*-*	*-*	*-*	*-*	*-*
Female	13	50	-	35	72↑	-	<0.001 *
Male	13	50	-	14	28	-	<0.001 *
*Experiment 2*	*-*	*-*	*-*	*-*	*-*	*-*	*-*
Female	13	50	-	35	70↑	-	<0.001 *
Male	13	50	-	15	30	-	<0.001 *
**Anthropometric data**	Mean ± SD	CI 95%	Min; Max	Mean ±	CI 95%	Min; Max	-
Age (years)	21.0 ± 2.3	20.8; 22.6	18.0; 28.0	70.5 ± 3.9↑	69.3; 71.5	63.0; 78.0	<0.001 *
Height (m)	1.73 ± 0.07	1.69; 1.76	1.56; 1.86	1.64 ± 0.08↓	1.62; 1.66	1.46; 1.84	<0.001 *
Body mass (kg)	67.6 ± 11.9	62.9–72.2	42.6; 90.5	73.7 ± 15.4	69.4; 78.0	42.5; 110.0	0.07
BMI (kg/m^2^)	22.6 ± 3.38	21.3–23.9	16.5; 30.4	27.3 ± 5.6↑	25.8; 28.9	17.1; 48.4	<0.001 *
Orthostatic Intolerance *	*n*	%	-	*n*	%	-	**-**
No	26	100↑	-	43	86	-	0.041 *
Yes	0	0	-	7	14↑	-	
**Number of cases**	*n*	%	-	*n*	%	-	**-**
Hypertension	0	0	-	20	40	-	<0.001 *
Diabetes	0	0	-	2	4	-	0.52
**Medication use**	*n*	%	-	*n*	%	-	**-**
Cardiovascular	0	0	-	20	40	-	<0.001 *
Psychotropic	2	7	-	7	14	-	0.47

Mean ± Standard deviation (SD); *n*: number of participants; 95% CI: 95% confidence interval; Min-Max: Minimum; Maximum; BMI: body mass index; ↓ lower than; ↑ Higher than. * Orthostatic intolerance symptoms were evaluated during the lie-to-stand transition, as this postural transition imposes higher orthostatic stress, resulting in a more pronounced cardiovascular adjustment due to the increased gravitational influence on blood volume distribution [1,28]. The grey colour represents the different sample characteristics displayed in Table 1.

**Table 2:** Comparison of time and frequency domain heart rate variability (HRV) analyses at baseline in the supine position between younger adults and older adults.

HRV (Time Domain)				
	Lie-to-stand		Stats	
	Younger adults	Older adults	*p*	ES
RR (ms)	888.7 | 316.6 (977.9; 1.01)	825.0 | 898.8 (999.7; 1.01)	0.213	−0.480
SDRR (ms)	63.9 ± 24.0 (54.1; 73.6)	36.0 ± 15.9↓ (31.5; 40.6)	<0.001 *	−1.462
RMSSD (ms)	77.7 | 51.2 (20.9; 141.0)	24.9 | 19.2↓ (7.2; 86.2)	<0.001 *	−1.758
**HRV (frequency domain)**				
	Lie-to-stand		Stats	
	Younger adults	Older adults	*p*	ES
LF (ms)	1060.0 | 1417.7 (95.8; 5352.0)	342.3 | 460.1↓ (31.4; 2226.0)	<0.001 *	−1.029
HF (ms)	2893.0 | 3984.7 (1.0; 9555.0)	402.5 | 640.0↓ (13.1; 5756.0)	<0.001 *	−1.322
LF/HF	0.4 | 0.2 (0.0; 0.8)	0.7 | 0.9↑ (0.1; 3.6)	0.002 *	1.306

|: Interquartile range with Minimum and Maximum; HRV: heart rate variability; ES: Effect size; RR: Average interval between consecutive heartbeats (RR-intervals); SDRR: Standard Deviation of RR intervals; RMSSD: Root Mean Square of Successive Differences between RR intervals; LF: Low-Frequency power; HF: High-Frequency power; LF/HF: Low-Frequency to High-Frequency power ratio: HR: heart rate. ↓ lower than; ↑ higher than. * Statistically significant differences. The grey colour represents the different HRV analyses displayed in Table 2.

**Table 3:** Comparison of the short-term cardiac baroreflex gain during sit-to-stand and lie-to-stand between younger adults and older adults across phases.

-	Sit-to-Stand				
Variable	Group	Phase 1 (30 s)	Phase 2 (60 s)	Phase 3 (180 s)	Phase 4 (420 s)
CBG (bpm.mmHg^−1^)	Younger adults	0.6 | 0.2 (0.4; 1.1)	0.6 | 0.2 (0.4; 1.1)	0.7 | 0.1 (0.5; 1.1)	0.7 | 0.2 (0.5; 1.0)
	Older adults	0.5 | 0.1 (0.3; 0.9)	0.5 | 0.1↓ (0.3; 0.9)	0.5 | 0.1↓ (0.3; 0.8)	0.6 | 0.9↓ (0.3; 0.8)
	**Adj *p***	0.10	0.0013 *	0.0013 *	0.0013 *
	ES	0.2	0.4	0.7	0.7
	**Lie-to-Stand**				
CBG (bpm.mmHg^−1^)	Younger adults	0.5 | 0.1 (0.4; 1.4)	0.6 | 0.1 (0.4; 1.2)	0.6 | 0.2 (0.5; 1.1)	0.7 | 0.1 (0.4; 1.2)
	Older adults	0.5 | 0.1 (0.3; 1.0)	0.5 | 0.1 (0.3; 1.1)	0.5 | 0.1↓ (0.3; 0.9)	0.5 | 0.1↓ (0.3; 1.0)
	**Adj *p***	0.6	0.06	0.002 *	0.002 *
	ES	0.07	0.3	0.5	0.7

|: Interquartile range with Minimum and Maximum; CBG: cardiac baroreflex gain; Adj *p*: adjusted *p*-value; ES: effect size. ↓ lower than. * Statistically significant differences between younger adults and older adults. The grey colours were used to represent the different postural transitions (dark grey) and groups (light grey).

## Data Availability

The raw data supporting the conclusions of this article will be made available by the authors on request.

## Abbreviations

ANS	Autonomic Nervous System
BP	Blood Pressure
BRS	Baroreflex Sensitivity
CBG	Cardiac Baroreflex Gain
CI	Confidence Interval
CO	Cardiac Output
CPM	Cardiac Parasympathetic Modulation
CSEP	Canadian Society for Exercise Physiology
DBP	Diastolic Blood Pressure
ECG	Electrocardiogram
ES	Effect Size
HF	High-Frequency Power
HR	Heart Rate
HRV	Heart Rate Variability
IQR	Interquartile Range
LF	Low-Frequency Power
LF/HF	Low-Frequency to High-Frequency Ratio
MAP	Mean Arterial Pressure
OA	Older Adults
OI	Orthostatic Intolerance
OH	Orthostatic Hypotension
RMSSD	Root Mean Square of Successive Differences
RR	R-R Interval (Interval between Heartbeats)
SBP	Systolic Blood Pressure
SAGER	Sex and Gender Equity in Research
SD	Standard Deviation
SDRR	Standard Deviation of RR Intervals
STROBE	Strengthening the Reporting of Observational Studies in Epidemiology
YA	Younger Adults
